# Interest in HIV Prevention Mobile Phone Apps: Focus Group Study With Sexual and Gender Minority Persons Living in the Rural Southern United States

**DOI:** 10.2196/38075

**Published:** 2022-06-13

**Authors:** Jeb Jones, O Winslow Edwards, Leland Merrill, Patrick S Sullivan, Rob Stephenson

**Affiliations:** 1 Department of Epidemiology, Emory University Atlanta, GA United States; 2 Department of Systems, Populations and Leadership, University of Michigan Ann Arbor, MI United States

**Keywords:** men who have sex with men, transgender persons, nonbinary persons, mHealth, mobile app, HIV, pre-exposure prophylaxis, PrEP, sexually transmitted infection testing, STI testing, HIV testing, mobile phone

## Abstract

**Background:**

Mobile health (mHealth) interventions, including smartphone apps, have been found to be an effective means of increasing the uptake of HIV prevention tools, including HIV and sexually transmitted infection (STI) tests and pre-exposure prophylaxis. However, most HIV prevention mHealth apps tested in the United States have been tested among populations living in areas surrounding urban centers. Owing to reduced access to broadband internet and reliable cellular data services, it remains unclear how accessible and effective these interventions will be in rural areas. In addition, gay and bisexual men who have sex with men and gender minority populations in rural areas experience enhanced stigma when compared with their more urban counterparts, and these experiences might affect their willingness and interest in mHealth apps.

**Objective:**

This study aimed to conduct online focus groups with men who have sex with men and transgender and gender diverse populations in the rural southern United States to assess their interest in mHealth HIV prevention apps and the features that they would be the most interested in using.

**Methods:**

Focus group participants were recruited from a larger pool of sexual and gender minority respondents to a web-based research survey. The participants indicated that they would be willing to participate in an online focus group discussion. Focus groups were conducted via secure Zoom (Zoom Video Communications Inc) videoconferencing. During the focus group discussions, participants were asked to discuss their experiences with HIV and STI prevention and how these experiences were affected by living in a rural area. They were then shown screenshots of a new app to promote HIV and STI prevention among rural populations and asked to provide their opinions on the app’s features. The transcripts of the discussions were reviewed and coded using a constant comparative approach.

**Results:**

A total of 6 focus groups were conducted with 26 participants. Most participants were cisgender gay and bisexual men who have sex with men (19/26, 73%); the remaining participants were transgender men (2/26, 8%), were nonbinary people (2/26, 8%), or had multiple gender identities (3/26, 12%). Participants reported numerous barriers to accessing HIV and STI prevention services and accurate information about HIV and STI prevention options. Overall, the participants reported a high degree of interest in mHealth interventions for HIV and STI prevention and suggested several recommendations for the features of an app-based intervention that would be the most useful for rural residents.

**Conclusions:**

These focus group discussions indicate that rural residence is not a major barrier to mHealth HIV and STI prevention intervention implementation and that there is a high degree of interest in these approaches to HIV and STI prevention.

## Introduction

### Background

The Ending the HIV Epidemic (EHE) initiative has identified 57 jurisdictions for increased HIV prevention resources [1]. Although many of these jurisdictions encompass urban centers, 7 states with large rural populations are identified as priority jurisdictions. Of these 7 states, 6 (Alabama, Arkansas, Kentucky, Mississippi, Oklahoma, and South Carolina) are in the southern United States and the seventh (Missouri) is geographically contiguous with the southern United States. These states were targeted because at least 10% of new HIV diagnoses in each of these states occurred in rural areas in 2016 and 2017. Similar to urban areas of the United States, gay and bisexual men who have sex with men (GBMSM) account for most of the new diagnoses in rural areas [2]. Despite the importance of rural communities in the EHE initiative, research is lacking on the sexual behavior and health care preferences of sexual and gender minority populations in rural areas.

Rural GBMSM are less likely to engage in important HIV prevention behaviors, including HIV and sexually transmitted infection (STI) testing and pre-exposure prophylaxis (PrEP) use [3,4]. They face increased and context-specific barriers to accessing HIV and STI prevention resources compared with men in more urbanized areas [5-7]. On the supply side, rural GBMSM are less likely to have access to culturally competent care [5-8], and PrEP awareness and lack of comfort in prescribing PrEP among providers have been barriers to PrEP uptake among rural GBMSM [7]. On the demand side, stigma and discrimination are barriers to engaging in HIV prevention. GBMSM in rural areas are less likely to disclose their sexual identity to their health care provider [9], report being afraid to seek health care, and avoid health care settings more frequently than urban GBMSM [10]. Experiences of stigma are heightened among rural GBMSM owing to more conservative attitudes toward same-sex sexual behavior and more insular social environments [11]. Intersecting minoritized identities intensifies these experiences among GBMSM of color [12].

Fewer HIV prevention studies have been conducted among transgender people living in rural areas; however, it is likely that the barriers to health care access experienced by transgender people in general [13,14] are exacerbated among transgender people living in rural communities. One study that included transgender women living in rural Florida found that transgender women were more likely to receive a late HIV diagnosis than their cisgender counterparts [15]. Transgender men in rural areas have been found to be less likely to have a primary care provider or to have had a blood cholesterol screening compared with urban transgender men [16].

Mobile health interventions (ie, apps) offer a potential solution to break down some of the barriers to HIV prevention services for rural sexual and gender minority individuals. Although data are not available for sexual and gender minority populations specifically, approximately 80% of rural adults use smartphones and 66% use social media [17,18]. In previous studies, GBMSM in rural areas have indicated that telehealth solutions for HIV and STI screening are acceptable methods for receiving these services [19,20]. Smartphone apps provide a discreet means of delivering sexual health information and can be a platform for delivering telehealth services, including HIV and STI testing and PrEP. Apps have been found to be acceptable to GBMSM [21], and a number of ongoing studies have assessed the efficacy of apps to increase HIV prevention services uptake among this priority population [22-26]. One app has shown efficacy in increasing the uptake of HIV testing and PrEP among higher-risk GBMSM [27]. However, studies testing the feasibility or efficacy of apps have tended to enroll populations recruited from urban centers or periurban areas. Men living in rural areas might hold heightened concerns regarding privacy and disclosure of sexual behavior or gender identity; these concerns might affect their willingness to use these apps [28]. Owing to these additional barriers faced by GBMSM and transgender people in rural areas, coupled with reduced limited access to high-speed internet and cellular services in some areas, data are needed on the feasibility and acceptability of app-based HIV prevention interventions for GBMSM and transgender people in rural areas.

### Objectives

We conducted focus group discussions with GBMSM and transgender and gender nonconforming people in the rural southern United States to assess health care use and willingness to use a mobile app to access sexual health information and order HIV or STI test kits to assess the feasibility of implementing app-based HIV prevention interventions in the rural southern United States.

## Methods

### Participants

Participants were recruited using web-based advertisements on Facebook, Instagram, Jack’d, Grindr, and Reddit and on social media feeds of community-based organizations serving rural sexual and gender minority communities. After completing a web-based eligibility screener, the participants completed a web-based survey and indicated their willingness to participate in an online focus group discussion. The eligibility screener and survey were only available in English. Eligible participants were aged 18 to 34 years; were cisgender men, transgender men, transgender women, and nonbinary people; reported ever having anal or vaginal sex; self-reported HIV negative; owned an iPhone or Android smartphone; and lived in a rural area of the southern United States, as defined by the US Census Bureau or the state of Missouri. Eligible participants were purposively sampled to obtain a diverse group with respect to age and race and ethnicity. More than half of all new HIV diagnoses in the United States occur in the South [29]. Missouri is geographically contiguous with the South and is a priority jurisdiction for the EHE campaign [1] because of the high burden of new HIV diagnoses occurring in rural areas of the state. Participants were compensated US $50 via an electronic gift card following the completion of the focus group discussion.

### Definition of *Rural*

Participants reported their ZIP code of residence, which was then mapped to the county of residence using an established algorithm [30]. Rural counties were those that were classified as micropolitan or noncore by the National Center for Health Statistics [31], had a Rural-Urban Commuting Area Code of ≥4 [32], or had an Index of Relative Rurality score of ≥0.4 [33]. None of these methods is designed specifically to categorize communities with respect to the availability of culturally competent care for GBMSM or transgender individuals, and it is not clear which of these schemes is most relevant to differentiating between rural and nonrural communities for this purpose. Thus, we used multiple definitions to have an inclusive criterion for participant eligibility.

### App

The Combine app is an adaptation of HealthMindr (Emory University in collaboration with Keymind and Softura) [21], a comprehensive HIV prevention app originally developed for cisgender GBMSM. The Combine app has the following functionality: frequently asked questions about HIV and STIs, PrEP, postexposure prophylaxis, and health insurance; recommendations for testing frequency; the ability to order HIV or STI self-test kits, condoms, and condom-compatible lubricants; behavioral risk self-assessments; and provider locators for PrEP and HIV or STI testing.

### Focus Groups

Focus groups were conducted on the web using Zoom (Zoom Video Communications Inc) and were recorded for transcription. Participants had the option of using a nickname and leaving their cameras off for anonymity. The focus groups started with a discussion about where participants usually accessed HIV and STI testing, experience with and interest in accessing HIV and STI testing on the web, experiences accessing health care and any issues encountered based on sexual or gender identity, and how living in a rural area affected their willingness and ability to access sexual health care services. Next, the participants were asked to describe the features of the smartphone apps that they liked and disliked. The participants were then asked to describe where they found information about sexual health (eg, on the web, from friends, or from health care providers). Finally, the participants were shown screenshots of the Combine app. After viewing the screenshots, the participants were asked to provide feedback on the app including interest in using the individual sections; willingness to order HIV and STI self-test kits, condoms, and lubricants through the app; whether they thought their peers would use it; and any functions that might be missing. Focus groups were stratified by gender identity so that cisgender men were grouped together and transgender persons, nonbinary people, and those with other gender identities were grouped together. Focus groups were conducted until saturation occurred overall but not necessarily within subgroups of gender identity (ie, no additional novel information was being generated). All the focus groups were facilitated by one of the coauthors (LM).

### Analysis

Transcriptions of focus group discussions were coded by 2 coders (LM and OWE) using a constant comparative approach [34]. Using this approach, coders first read the transcripts and identified broader emergent codes, which were used to construct an initial codebook. After an initial review of the multiple transcripts, the coders came together to discuss and clarify the meaning of each code. Subsequently, codes were applied to all transcripts, and newly emergent codes were added to the codebook. After this full pass, the coders once again met to probe the meanings of codes and to define higher-level themes that were emerging. In addition to the emergent themes that were identified, the a priori themes of anticipated and enacted stigma were included. Following this meeting, all transcripts were coded a second time using the finalized codebook.

### Ethics Approval

The participants provided informed consent to participate in focus group discussions. All study procedures were reviewed and approved by the Emory University Institutional Review Board (protocol 00001268).

## Results

### Participant Demographics

A total of 91 participants (77 cisgender men, 8 nonbinary people, 2 transgender men, and 4 with multiple gender identities) were eligible and expressed willingness to participate in the focus groups. Of these 91 participants, 26 (29%) ultimately agreed to participate and contributed to 6 focus groups, comprising 2 to 8 participants per group. The demographic characteristics of the participants are presented in Table 1. Most participants (19/26, 73%) were cisgender GBMSM; the remaining participants were transgender men (2/26, 8%), nonbinary people (2/26, 8%), or had multiple gender identities (3/26, 12%).

**Table 1:** Demographic characteristics of focus group participants (N=26).

Demographics				Value
Age (years), median (IQR)				25 (21-29)
**Race or ethnicity, n (%)**				
	Asian participants			1 (4)
	Black participants			6 (23)
	Mixed race participants			18 (69)
	White participants			1 (4)
	**Hispanic participants, n (%)**			
		Yes	1 (4)	
		No	25 (96)	
**Sex assigned at birth, n (%)**				
	Male			22 (85)
	Female			3 (12)
	Intersex			1 (4)
**Gender identity, n (%)**				
	Cisgender men			19 (73)
	Transgender men or transmasculine			2 (8)
	Nonbinary or gender nonconforming			2 (8)
	Multiple identities			3 (12)
**Education, n (%)**				
	High school or GED^a^			3 (12)
	Some college			10 (38)
	College graduate			13 (50)
**Insurance, n (%)**				
	Private			20 (77)
	Public			2 (8)
	Other			2 (8)
	None			2 (8)
**Annual income (US $), n (%)**				
	<19,999			3 (12)
	20,000 to 39,999			5 (19)
	40,000 to 74,999			7 (27)
	>75,000			7 (27)
	Prefer not to answer or do not know			4 (15)

^a^GED: General Educational Development test (an alternative to a high school diploma in the United States).

### Focus Group Discussions

Qualitative data analysis identified 5 major themes, which are described in the next sections, with representative quotations from participants.

#### Access to Health Services

Participants talked about access to health services both in terms of their ability to access health care directly and in terms of access to transportation and internet services to facilitate the uptake of health care services. Participants often reported that accessing lesbian, gay, bisexual, transgender, and queer+ (LGBTQ+)–competent health care was not always possible in the smaller towns where they lived:

I’d say I’d probably have to travel about an hour to the nearest big city that’s accepting...’Cause my area is not exactly the most accepting, in that regard.

Others have described issues where STI testing might only be available on certain days in their community if not ordered through a primary care provider. In contrast, some participants described being able to access sexual health care in their jurisdiction from an LGBTQ+-specific health center, a primary care provider, or some other sexual health provider:

Luckily, where I live, there’s kind of a specific clinic for...I think it used to be called [name of organization], but they’ve kind of transitioned into a more comprehensive HIV prevention and care place. So they are, I think, probably the main providers of PrEP and STI services here.

For those who did not have an accepting clinic in close proximity, access to transportation could facilitate or limit the use of LGBTQ+-competent health services in larger cities:

Well, at least for me, because of my disability, transportation issues is a major factor, as well. There are no Planned Parenthoods in my area, and the only health organization that would otherwise do STI testing is through the closest university, which is still like 25 minutes away, so it’s not very fast.

Another participant noted that there were providers available locally, but the fear of being outed made them undesirable. Thus, transportation was a barrier despite proximity to the available providers:

When I was younger, it was more of a barrier, because it’s a small town, so all of my...All the providers kind of knew me and my family, so that was definitely...And no way of travel, was a barrier...Something that was very anxiety-provoking, when I needed...I knew that I maybe needed a test, but then going to access one was kinda traumatic, so I avoided it sometimes.

#### Cultural Environment

*Cultural environment* described the experience of living in small, rural communities wherein privacy is limited, both inside and outside of the household, and socially conservative values are prevalent. Participants consistently described town dynamics where community members were curious about one another’s private lives and privacy was difficult to maintain. To avoid gossip, this participant chose to use at-home testing instead of seeking sexual health care in person:

I go to a Christian college, so it’s kind of difficult around the area to go in for screenings, just because it’s kind of a small town as well, everybody talks, so it’s not very welcomed.

As demonstrated by the following participant, a common concern was that one’s sexuality could be revealed to the community as a result of using sexual health services or products:

Well, because when you have to go to the store for those things, you gotta go in person and so...I don’t know a whole lot about straight sex, but I’m sure they’re not all buying lube and...So you went and bought lube and now everybody knows that you’re [chuckle] doing something.

In addition to this lack of privacy, participants described navigating socially conservative values and stigma toward LGBTQ+ people, both in the community and in health care contexts. A major concern was the lack of relevant sexual health knowledge, which participants traced back to inadequate and stigmatizing sexual education experiences in school:

Growing up, I went to a religious high school and sex education was nonexistent and my parents didn’t really teach me anything. In my biology class, I mean it said things like “erection” and “clitoris” and things like that. The school actually glued the pages together in our textbook, so we couldn’t see them.

Conservative values and stigmatizing views were also encountered in medical settings. Many participants described experiences where staff and providers were tangibly uncomfortable working with them:

Honestly, here where I’m from, Arkansas, they’re just...Everything is kinda backwards in here. It’s like as soon as they figure out that you’re of the LGBTQ community, they almost have like a step back, “Oh my God.” It’s weird.

I think I face more stigma because I’m in the sex work industry, and is going to a PCP is definitely hard to explain why I need to get tested so often. And then there’s just that stigma surrounding that, it just makes it incredibly uncomfortable.

Other participants described anticipating stigma when seeking medical care:

Well, I just moved, like I said, and I haven’t yet found a PCP. And so, I would have to get over that barrier once more, like fear of judgment from a PCP, so I’m trying to find a new one, and right now I’m going to Planned Parenthood.

As the following participant explains, although doctors are expected to be professional and exercise confidentiality, they are also entrenched in the community and sociopolitical milieu in which they live and work:

I had a primary care physician that I shared with my immediate family for...‘Cause I’m from a very small town so there’s not a lot of options. And once I came out, it just felt a little bit different ‘cause I know...It’s very small so everybody knows everybody and people are doctors, but they’re also just people in your community as well. So, once I actually went to college and graduated out of my parents’ insurance and got my own insurance, I didn’t really feel the need to continue to stay at that same place.

#### Discretion or Confidentiality

Participants in all the focus groups frequently emphasized the necessity of *discretion* in the form of privacy and confidentiality when it came to seeking or accessing sexual health services. These concerns were particularly heightened because of the lack of privacy and conservative values implicit in the *cultural environment* and the potential for shared familial housing arrangements:

But yes, especially a sense of discretion, if we’re talking about queer people in rural areas. I know my hometown, my one-stoplight hometown, if anybody found out it would be a huge shunning issue in the town.

For participants who may not be out to their local community or family, confidentiality is not only necessary to maintain their safety and social standing but also a prerequisite for their willingness to access sexual health services. Participants needed to know that their information would be kept confidential and that discretion would be prioritized, whether by local health care providers or by an app. One participant relayed an experience where confidentiality was compromised:

And while I consider what happened as coincidental or incidental, [a clinic employee] actually outed me and that really changed the dynamic of my whole life...It’s sort of like having a conversation, “Hey [name], I saw...Oh yeah, you came down to XYZ Clinic.” And everyone knows that clinic to be a specific clinic for the LGBTQ+...And I was like, “Oh.” And you can probably follow the line from there.

Owing to concerns about discretion when accessing care in person, some participants preferred to use telehealth and home testing offered by services such as Mistr, a web-based sexual health provider that caters to the LGBTQ+ community [35]. The participants identified the ability to receive confidential testing and care from the privacy of their own space as a major benefit of telehealth.

When discussing apps, the participants felt that discretion should be implemented at all levels, from the visual branding of the app to the app’s privacy policies to the need to identify themselves within the app:

I know certain apps that used levels of discretion will name it something else or even have the ability to change what icon it is. I’ve seen things where you don’t necessarily want someone to know you have an app of X, Y or Z, so it’ll show up as a calculator or something like that on your phone. If somebody is looking over your shoulder and you’re thumbing through your apps, if you have someone nosy, they don’t see something specific for it, it just looks like another calculator app, or mix up possibly the ability to change the icon or something along that line, or maybe an abbreviation that only a few people would understand to maybe help with a level of discretion.

One participant suggested that the app be password protected, which would help youth and others whose phones may be checked by family members keep their information private. When it came to marketing the app and the mailing of sexual health tests or supplies, discreet packaging was recommended to help maintain the privacy of the recipient:

I think the only thing I could really think of is just making sure that the materials were sent in a very discreet box. I live in a very small town and everyone knows each other, including the mailman, so...I think it being discreet is critical.

...If you’re living with your parents, maybe not the most great thing for them to see on their doorstep. But if you are living alone or living elsewhere, I think, at that point, it’s okay with discretion. I think it’s just better to be safe and more discreet than sorry, in a way.

Packaging that maintained the recipient’s privacy was very important, especially for those who might be living in multigenerational households. Finally, for transgender and nonbinary participants, privacy was also extended to the name used in the packaging. In some housing situations, transgender and nonbinary participants may need to use their birth name for safety, whereas in other contexts, they may be able to use their chosen name. Having the option to specify which name to use when ordering sexual health tests or supplies was a way of maintaining *discretion* around one’s gender identity:

I also think the name thing, when you go to order an item, you can do your preferred name or the legal name or whatever, and have that option right in front of you. Because if you’re living one place, one time, and you can use your preferred name when you want your orders with that, or if you’re living in another place, you need it as the other name, whatever that may be.

#### Convenience

In rural settings where culturally competent health care providers may be located far away and transportation may be inaccessible, *convenience* was paramount to accessing sexual health services and materials. In the context of sexual health services and supplies, *convenience* referred not only to ease of use but also to timeliness, affordability, and ease of attainment. A participant described the convenience of home testing compared with visiting a provider:

I do like the ease and convenience of using a service like Mistr. And they do have a referral program, and I’ve sent it to a few of my friends just because I’m getting [PrEP] for free, and it’s very easy. It’s delivered to me. I don’t have to go to Walgreens and have them tell me that they don’t have my prescription that day, ‘cause it’s on back order or whatever...I like the immediacy.

When sexual health care was not convenient, whether it took too much time to access or was hard to afford or attain, participants expressed that they might be more likely to put it off:

I was looking because I didn’t want to have to travel and take time out of the day to go make an appointment and find somewhere. Where I live, you can only do STI testing on certain days if you don’t go to your PCP. I just thought it’d be more convenient to do it at home, but my insurance wouldn’t cover it, and it seems like the cost, I was in law school at the time, it was somewhat cost-prohibitive, so I just ended up submitting late.

Participants expressed eagerness to use sexual health care options they deemed convenient, and this followed through to their interest in the app. Considering the app, many participants appreciated that it was a *one-stop shop* for HIV testing, sexual health products, mental health screenings, and locating providers:

I think that it’s gonna be potentially super good for...Especially for people in rural areas, not just the convenience and the discretion, but just even having the access to those services. And the convenience of an app and having it all in one area is awesome.

When speaking about the locators in the app that allow users to find HIV or STI testing, PrEP providers, and mental health and substance use treatment providers in their area, users thought that these features would be useful in identifying conveniently located providers:

I love this idea [locator], mainly because it’s convenient, not a lot of people know where they can get PrEP and information on PrEP or things like that, so this is really convenient.

Participants were also very interested in low-cost or free services through the app, given that affordability and insurance status could be barriers:

I think if there’s funding for some of those resources to be mailed out for free, that’s probably the most, I would say, really beneficial aspect.

Another participant shared how a sexual health app would benefit him:

Right now, I don’t have a means of transportation, and so having something like this [app], where I can have things delivered or I can chat with a professional over the phone, I find that really convenient.

Thus, participants viewed the app favorably and perceived it to reduce various barriers to care.

#### Trust or Comfort

*Trust* and *comfort* came up in 2 primary contexts: when ascertaining the quality of health information and when considering one’s relationship with a health care provider. When it came to information, participants wanted content that they deemed to be accurate, legitimate, and trustworthy. Many participants were very discerning when evaluating health information and checked multiple sources to ensure that information was factual:

I’d like to check out a few different sources just because I want to get the most accurate picture of today’s standard practices, and if anything new has been found out...Yeah, I’ve looked at WebMD, Healthline...Planned Parenthood’s website is a good resource. Yeah. Usually those...Also, I like to read white papers. If I need to find something out, I’ll look it up on PubMed or something else too.

Often, the origins of health information were considered when determining whether content was factual; that is, participants were more inclined to believe health information that came from a trusted friend or website than information they might find through a search engine or social media:

If it’s coming from the Kaiser Family Foundation, I know that I can trust it just because they have epidemiologists and PhDs and experts in public health that are providing that information, but if it comes from Fox News and it’s not cited, questionable.

I have a few friends that if I have any kind of need for any kind of...Not just necessarily to search for help, but in general. I can just be like, “Hey, do y’all have any experience with this?” and if they tell me no, then I’ll probably go to Google or just ignore it until it becomes a bigger problem.

If a source was trusted, a participant would feel *comfortable* or willing to use that source. When it came to providers, *trust* was also intertwined with *comfort*, an understanding that a provider would be accepting of one’s identity, provide or foster a feeling of safety, and be able to provide LGBTQ+-competent care:

Honestly, I’d feel really comfortable with finding another LGBT resource center and something specifically for people like me. So, if...Not really like Planned Parenthood, but something like that, I’d feel really comfortable in an environment like that.

For participants, it was important to locate a provider who could offer caring, LGBTQ+-competent services. On top of that, building a close working relationship with a provider was conducive to high-quality care. On the flip side, some participants struggled to locate providers that were accepting, which was partially attributed to the issues of *cultural environment* and stigma:

I think for me, I get the primary care physician being the...I can see why that’s more desirable. I think for me, I just don’t know how to go about finding somebody that I would feel comfortable with...Especially, I think maybe as [other participant] mentioned, like small towns and stuff like that, that stuff’s really hard there, I think.

This participant also identified moving frequently and aging out of pediatric care as contributors to the struggle to find a trusted primary care provider. As a student who was about to graduate, they were not sure what they would do for sexual health services upon graduating. Other participants were able to access LGBTQ+-specific providers but felt pigeonholed and uncomfortable with their approach to treatment:

Well, going there, going to my primary care physician, they don’t see me as a gay, bisexual male. But when I go to a health center, they treat me as...It’s sort of like, “Oh, a gay person is here so we’re gonna do this.” And the type of service that I receive from them is completely different than the type of service and care that I receive from my primary care physician where I use my insurance.

For this participant, it was important to be viewed holistically and not simply through the lens of his sexuality. Therefore, he had switched from a community health center to a primary care provider. When participants did not feel *trust* or *comfort* in a provider, locating and using health services were major concerns:

I think that one of the things that, before I even use the app, we were talking about credibility earlier, and I think that I would trust the information on this app, if either there were sources or when you open the app, it’s like it says the name of the app, but it’s like powered by or created by Emory or some university. I feel like that would give it some credence.

### Preferences for a Mobile HIV and STI Prevention App

#### Overview

App features describe feedback directly pertaining to what participants were looking for when accessing sexual health information and care in a digital environment. Many of these responses were elicited in response to discussion of mock-ups from the Combine app or in discussion of other apps and web-based resources that participants use frequently. Important features included *relevant information* and *functional recommendations*.

#### Relevant Information

Participants expressed a desire for information that was relevant to their experiences as members of the LGBTQ+ community when searching for sexual health information, whether in person, on the web, or through a health app. It was important for participants to feel that the health information and care they received were tailored and *relevant* to their needs as an LGBTQ+ person.

Given that many participants discussed receiving inadequate sexual education, they were very interested in having a detailed frequently asked questions section that would provide accessible information about options for safe sex and navigating consent, including introductory topics:

I don’t know exactly how this could fit into the app, but when talking about sexual health, just...Yeah, even like the condom use page, maybe if there was even like a diagram, I mean even with something like a dildo or whatever, “This is how you put a condom on.” Because I never was shown how to use one. I haven’t had a ton of sexual partners, but I’ve actually never used one, and so that would have been really nice to know or other options for that.

Another participant added:

In addition, to just safe sex practices, also just things like healthy relationships and consent, and that sort of stuff, I think that could be really beneficial, especially for young people in rural areas....

For transgender, nonbinary, and bisexual or pansexual participants, it was important to have information that was applicable and affirming to their gender identity and anatomy and the anatomy of their sexual partners:

Yeah, I think it was on Gilead’s website, or maybe it’s on aids.gov that they ask you what your gender identity is, what your anatomy is, and then they ask you a question about the anatomy and gender identity of your partners. So, that way they can customize the suggestions for you, so I don’t know...That would be like the first thing when you create a profile at the first time you open the app, and then if you need to change it, you can hit the wrench or the gear and change it another time.

Ultimately, participants were ready to engage with relevant, tailored information about sexual health.

#### Functional Recommendations for HIV and STI Prevention Mobile Apps

Participants provided various *functional recommendations* about features that could be added to the app to improve its usability and better serve users’ needs. Many participants wanted the app to integrate easily with existing utility apps on their phone. For example, one feature of the Combine app is a timeline that displays study milestones and planned prevention activities (eg, scheduled HIV tests and reminders to order condoms). One participant noted that they would want to integrate this feature with other reminders on their phone:

I really like the timeline thing, which is I know that if I don’t have things in front of me, they don’t exist. I think it would also be a really useful feature to have a calendar integration, add the dates to your calendar. But I also really like how you can see all of the different steps that you have to participate in. That was a feature that I really appreciated.

Participants were interested in the ability to track shipments of sexual health materials or testing kits ordered via the app:

I think that there’s something about being able to monitor and track something in real-time is very gratifying, and if you can ever...I feel like I ever have a service that allows me to do that, it kinda makes me wanna use it more. One of the reasons I have shipped through FedEx is because their online tracking is so solid. I think just having the ability to kinda track and feel like you’re in constant touch with whatever it is you’re trying to do is a really nice thing to have.

Participants wanted the ability to easily tailor their search to locate content within the app, whether sexual health information or providers that offer specific services:

I think of it like Zillow, where if you wanna just look at properties, you can look at properties, but then you can filter based on like condo or house. I like that it has a list of options in the geographic location. I don’t know if every center has testing, screening, if hopefully, they’re all queer and trans-friendly. But I don’t know that, I’m just assuming things like the MinuteClinic is gonna have a different kind of culture than if you go to a sexual health center.

Adding improved search features would improve the app’s ease of navigation and *convenience* for users. When discussing the mental health and substance use self-evaluation tools available on the app, participants wanted the app to link them to care when necessary:

I was gonna say it might be really helpful to have it directly linked to you where you can go through the diagnostic process. So, if you were tested with the depression or whatever, and you’re like, “Okay, well, what can I do to get better? How can I help this?” If it had the tips, the infographic, self-help tips, you can do that, or you can be like, here’s a psychiatrist you can go to if you want access to medication if it’s really not going well for you. Because I really had no idea where to start with that when I got diagnosed with BPD and stuff. I had no clue what to do.

To better help them use the provider locating feature, participants were interested in a rating and review system in which they could report their experiences with providers. This was perceived as a potentially effective method to avoid stigmatizing health care experiences.

Maybe after you set an appointment, there’s a questionnaire and you say your experiences with that certain facility. I feel like if people within the app that have been there and use it, they know exactly what they were getting into.

Another recommendation that came up across different focus groups pertained to internet *access* in rural areas. Dedicated offline functionality would make the app more usable and *convenient* for individuals with poor or fluctuating internet connections:

This might sound a little bit weird, but a very good/dedicated offline feature, at the very least for the FAQS and stuff like that, and maybe even the quizzes. ‘Cause a lot of rural areas don’t have the best access to internet. Even with mobile data, sometimes it’s difficult to get a good connection and things can fail.

## Discussion

### Principal Findings

We sought to understand the facilitators of and barriers to the uptake of sexual health services among sexual and gender minority individuals living in the rural southern United States and their interest in and willingness to use a mobile app to access HIV and STI prevention information and telehealth services. In focus groups with cisgender men who have sex with men and transgender and gender-expansive populations in the rural southern United States, participants reported frequent barriers to receiving appropriate sexual health services and a high interest in telehealth and, specifically, mobile apps to access HIV or STI prevention information and services.

Participants described a variety of experiences with health care providers, with most participants reporting stigmatizing experiences. Although some participants were able to access clinics that specifically cater to the LGBTQ+ population, many had to travel substantial distances to these clinics. PrEP providers have been documented to be clustered around urban areas, with many people in rural areas living in PrEP deserts that require travel of 1 hour or more to reach a PrEP provider [36]. This lack of proximity to needed HIV prevention services highlights the need for alternative methods for accessing health care, including telehealth, which has been found to be acceptable to rural GBMSM [19,20].

Participants held overwhelmingly positive views of the Combine app and expressed a high degree of willingness to use it to access HIV or STI testing and other HIV prevention services. The app was viewed as an efficient and effective method for overcoming the several barriers to accessing sexual health care discussed earlier. Although some of the barriers to living in rural areas are shared, GBMSM and transgender and gender-expansive communities are not homogenous. Interventions that have been found to be acceptable to GBMSM in densely populated areas, such as the several HIV prevention apps currently being tested, might not be transportable to rural communities. Our results indicate that app-based interventions might be transportable, but adaptations might be necessary to make them acceptable. Although discretion is always a concern when planning interventions for marginalized communities, the concerns of rural sexual and gender minority individuals are heightened because of the increased insularity of the broader communities in which they live. Indeed, rural men who have sex with men have been found to have concerns about privacy and confidentiality in technology-based HIV prevention research studies; however, these concerns were not perceived as insurmountable barriers to participation [28]. In addition, participants indicated a lack of access to basic information about sex and sexual health care such that additional information and resources might be necessary to include.

Drawing on their experiences of living in rural areas, participants had several suggestions for content that should be included in the app. For example, participants discussed the lack of relevant sexual health education that LGBTQ+ students in rural areas receive, a barrier that has been noted elsewhere [37]. To compensate for this, participants suggested that an app for rural sexual and gender minority users should include basic information about sex, the risk of HIV and STIs, and options for reducing the risk of HIV and STI transmission. Participants highlighted that multimedia presentations of this information (eg, diagrams or animations of how to properly use an external condom) would maximize its utility. Inclusive language with respect to anatomy was also mentioned as a priority. This highlights the potential need to include responsive design elements that would allow a user to specify their preferred terminology for different parts of their anatomy (eg, front hole, vagina) upon first use so that language can be used appropriately in the app.

Participants also had several functional recommendations to improve the app, both in general and to increase its utility for rural users. Participants wanted the Combine app to be able to integrate with other apps on their phone. Currently, Combine integrates mapping apps to provide directions to testing locations identified within the app. However, Combine also includes a feature that allows users to set one-time or recurring reminders for different prevention activities (eg, HIV or STI testing and ordering condoms). Participants suggested that integrating this feature with calendar apps on their phone, which they use more frequently, would make this feature more valuable. Future adaptations of the app should incorporate as many app integrations as feasible to increase the utility of the Combine app. The suggestion to make most of the app’s functions available offline would also increase its utility for rural residents with poor cellular coverage.

Participants expressed multiple motivations and methods that contributed to their decision to trust a source of information. The lack of advertising on a provider’s website was perceived to legitimize their services by demonstrating that they were not dependent on external support. Similarly, affiliations with trusted organizations, such as research institutions, were perceived to confer legitimacy. Implementers should consider the extent to which endorsements from or affiliations with existing organizations might improve buy-in from potential end users.

Participants also wanted to be able to search for content within the app. Referencing a real estate app, one participant indicated that they would be able to find the information they wanted more quickly if they could search or use different filters to narrow down the information presented to them. This type of feature could be particularly useful for experienced users who revisit the app to search for particular information.

A requested feature that participants suggested would be particularly useful for sexual and gender minority users in rural areas is a ratings and review system. To supplement the provider locator within the app, participants wanted the ability for users to rate providers and write reviews of their experiences. These ratings could be implemented in several ways. For example, ratings could be provided based on the overall experience or for certain aspects of the experience (eg, aspects of culturally competent care). Ratings could also potentially be presented based on the identity of the user providing the rating (eg, cisgender gay men and transgender women) so that other users could view the ratings most relevant to their own experience. Implementing this type of system, however, would require a substantial amount of effort to moderate, and rules would have to be generated for when to display user-submitted ratings and reviews; for example, after a certain number of ratings have been received for a given provider.

### Limitations

These focus groups were a convenience sample of sexual and gender minority participants recruited via the web. Thus, they are not representative of all sexual and gender minority rural residents in the southern United States. Willingness to use a mobile HIV prevention app among those who are or are not online and do not volunteer to participate in research studies might differ from those who do. Participants also did not have the opportunity to interact with the Combine app; therefore, they were unable to provide detailed feedback on its specific functionality.

### Conclusions

The results of these focus group discussions indicate that sexual and gender minority individuals in the rural southern United States could benefit from HIV prevention interventions delivered via mobile apps and that there is high interest in and willingness to use such apps. To meet the goal of the EHE initiative, all communities at risk of HIV must have access to HIV prevention and treatment services. Mobile apps might present an effective and scalable method for reaching sexual and gender minority individuals in rural areas, as they have already been shown to do in more densely populated locations [21,38,39].

## Abbreviations

EHE: Ending the HIV Epidemic
GBMSM: gay and bisexual men who have sex with men
LGBTQ+: lesbian, gay, bisexual, transgender, and queer+
mHealth: mobile health
PrEP: pre-exposure prophylaxis
STI: sexually transmitted infection
